# Convolutional Neural Network-Based Machine Vision for Non-Destructive Detection of Flooding in Packed Columns

**DOI:** 10.3390/s23052658

**Published:** 2023-02-28

**Authors:** Yi Liu, Yuxin Jiang, Zengliang Gao, Kaixin Liu, Yuan Yao

**Affiliations:** 1 Institute of Process Equipment and Control Engineering, Zhejiang University of Technology, Hangzhou 310023, China; 2 Shanxi Key Laboratory of Signal Capturing & Processing, North University of China, Taiyuan 030051, China; 3 Department of Chemical Engineering, National Tsing Hua University, Hsinchu 300044, Taiwan

**Keywords:** flooding detection, non-destructive evaluation, deep learning, convolutional neural network, image processing, classification, packed column

## Abstract

In chemical processes, packed columns are frequently employed in various unit operations. However, the flow rates of gas and liquid in these columns are often constrained by the risk of flooding. To ensure the safe and efficient operation of packed columns, it is crucial to detect flooding in real time. Conventional flooding monitoring methods rely heavily on manual visual inspections or indirect information from process variables, which limit the real-time accuracy of results. To address this challenge, we proposed a convolutional neural network (CNN)-based machine vision approach for non-destructive detection of flooding in packed columns. Real-time images of the packed column were captured using a digital camera and analyzed with a CNN model, which was been trained on a dataset of recorded images to identify flooding. The proposed approach was compared with deep belief networks and an integrated approach of principal component analysis and support vector machines. The feasibility and advantages of the proposed method were demonstrated through experiments on a real packed column. The results showed that the proposed method provides a real-time pre-alarm approach for detecting flooding, enabling process engineers to quickly respond to potential flooding events.

## 1. Introduction

Packed columns are a type of gas–liquid mass transfer equipment commonly used in the chemical industry for processes such as distillation, gas absorption, and liquid-liquid extraction. They are favored for their simple structure, low operating costs, and versatility in using different types of packing materials. However, the performance of packed columns, specifically in terms of energy efficiency and capital investments, is crucial to the overall production economy. Conventional packed columns consist of a vertical cylinder filled with packing materials. In a counter-current operation, gas enters the column from the bottom and flows upwards while liquid enters from the top and exits through the bottom. The goal is to increase interaction between the liquid and gas to maximize productivity. However, the packing materials can reduce the cross-section for the free flow of gas and liquid, leading to flooding if the flow rate exceeds a certain limit [1,2]. This can negatively impact the performance and energy efficiency of the process, and even damage the production system. Thus, there is a need for real-time, efficient, and accurate flooding detection methods.

Conventional methods for detecting flooding in packed columns primarily include manual visual inspection, liquid holdup measurement, and pressure drop monitoring. These methods have limitations, such as the poor accuracy and reliability of visually based inspections, the disruption to operations caused by measuring liquid accumulation, and the use of indirect information. Additionally, many of these methods are unsupervised learning techniques, which may not be as accurate as supervised methods. Therefore, it is essential to develop more accurate and reliable flooding detection methods based on computer vision techniques. The literature indicates that flow structures associated with the onset of flooding can be observed visually through a transparent column or observation window [3], but the reliability of this method is limited. Flooding can also be identified by measuring the amount of liquid retention in the column [4], but this method requires stopping the flow of both gas and liquid, making it unsuitable for online monitoring. Additionally, pressure sensors are commonly used for flooding detection [5,6,7,8] because a dramatic change in pressure signals can be observed when flooding occurs. However, machine vision-based non-destructive detection methods are still lacking in the literature, and it is necessary to develop such methods.

In recent years, machine learning-based data mining and analytics have emerged as promising solutions in process industries [9,10,11,12,13,14,15,16,17]. Some methods have been successfully applied in the task of flooding monitoring in packed towers. Brockkötter et al. [2] developed a Gaussian process-based data-driven model to predict the flooding state of filled liquid-liquid and high-pressure extraction towers. They tested the model’s performance under different chemical systems and unstructured packing geometries, using various Gaussian process regression algorithms. In another study, the same authors developed a grey-box data-driven model that incorporates domain knowledge, demonstrating its ability to accurately derive and interpret repulsion profiles for various extraction towers. Additionally, Oeing et al. [8] applied machine learning algorithms for flood detection in laboratory distillation and liquid extraction columns. The results indicate that both process-valued time-series data and image recognition can be effectively utilized for modeling purposes.

Convolutional neural networks (CNNs) [18], as one representative deep learning method, are a key focus for solving computer vision problems. Specifically, CNNs have proven to be highly effective in extracting features from images or videos [19,20,21,22,23]. Their local connectivity and weight-sharing structure make them ideal for image recognition and classification. To the best of our knowledge, the application of CNNs or similar techniques for detecting flooding in packed columns remains an uncharted area of research. This work aimed to advance the success of convolutional neural network (CNN)-based machine vision in the real-time and efficient detection of flooding in packed towers, as opposed to traditional flooding detection approaches that primarily rely on human vision and selected process variables. The key contributions of this study are:

1. We introduced a deep learning-based machine vision approach using CNNs for non-destructive detection of flooding in packed columns. Different from the results presented in the previous literature [8], which mainly focused on classification, the proposed method offers a real-time pre-alarm approach for early detection of flooding;
2. Real-time images of the packed column were captured using a digital camera and analyzed through a pre-trained CNN model. This approach, based on a dataset of recorded images, enabled the prediction of flooding and provides process engineers with a timely indication of potential flooding occurrences;
3. Additionally, we also evaluated an integrated approach combining principal component analysis (PCA) [24] and support vector machine (SVM) [25], as well as a deep belief network (DBN) method [26], for flooding detection. These experiments were conducted on a real packed column and demonstrate the feasibility and superiority of our proposed approach.

The remainder of this work is organized as follows: Section 2 presents three visual inspection methods for flooding identification, Section 3 describes the experimental system, Section 4 presents the application results and discussions, and finally, conclusions are made in Section 5.

## 2. Methodology

### 2.1. CNN Method

The concept of CNN is a biologically inspired variant of the conventional multilayer perceptron [27]. In recent years, CNNs have been validated to be highly effective in image recognition and classification [9,13,28,29,30]. It is natural to consider using the CNN algorithm to identify the flooding phenomenon from the column monitoring images collected by the camera.

**Figure 1:**
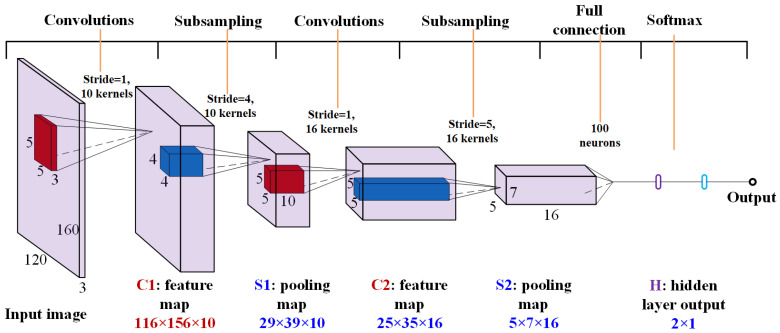
The architecture of CNN for flooding identification.

The architecture of the CNN used in this work is illustrated in Figure 1. This network contains two convolutional layers each of which is followed by a pooling layer. A bias term is added to each convolutional layer, while average-aggregation is adopted in each pooling layer. Then, a fully connected layer is used to combine the features extracted by the previous layers and feed the vectorized feature maps to the Softmax classifier. More detailed description of the model structure will be introduced in Section 4.

The core of a CNN is the convolution layers. In the network, each unit of a convolution layer only receives inputs from a set of units located in a small neighborhood in the previous layer and calculates the output features using a number of filters (also known as kernels) with assigned weights. Such a neighborhood is called a local receptive field. In a CNN, each local filter is replicated across the entire visual field. Therefore, the outputs of each filter form a feature map. Typically, the input of a convolutional layer is the original image, or a set of feature maps outputted by the previous layer, which is commonly considered as a volume with size of *m* × *n* × *r*. Here, *m* and *n* denote the height and width of the image, or the feature maps inputted to the layer. For the first layer, *r* is the number of channels of the original image. For example, an RGB color image usually has *r* = 3, where the three channels correspond to red, green, and blue, respectively. For the other convolutional layers, *r* is the number of the feature maps. Denote each channel or each feature map inputted to a convolutional layer as **I***^i^*, *i* = 1, …, *r*, and suppose that this convolutional layer has *k* kernels each of which usually has a size of *c* × *d* × *q*, where *c* and *d* are smaller than the corresponding dimensions of the input image, i.e., *m* and *n*, and *q* can either be equal to *r* or smaller and may vary for different kernels. The output feature maps **O***^s^* of this layer, *s* = 1, …, *k*, are then calculated as:

(1) $$O^{s}=\sum {}_{i=1}^{q}(W_{i}^{s}*I^{i}),$$

where * is the convolution operator, *s* is the filter index, and **W** denotes the kernel. Therefore, the size of the output of a convolutional layer is (*m* − *c* + 1) × (*n* − *d* + 1) × *k*.

In a CNN architecture, it is common to insert a pooling layer between two successive convolutional layers. After pooling, the aggregate statistic over the regions of the convolved feature space is calculated, which greatly reduces the spatial size of the feature maps. Hence, the amount of parameters and the computational burden is reduced, while the over-fitting is controlled. The most popular aggregate statistics used in CNNs include maximum value and average, which are calculated as the maximum value and the mean value of the numbers in the corresponding regions, respectively. Usually, an additive bias is applied to each feature map either before or after the pooling layer.

In addition, it is noted that the convolutional and pooling layers introduced above only lead to linear transformations over the input data, which are not sufficient when dealing with complex nonlinear data characteristics. To solve this problem, a nonlinear activation function should be used, which is an element-wise operator. The activation function takes the feature map generated by a convolutional or pooling layer as its input and creates the activation map as the output. Therefore, the input and output of an activation function have identical dimensions. The rectified linear unit (ReLU) [31] is the most commonly used activation function in the recent CNN architectures, which is a piecewise linear function that is defined as:

(2) f(u)=max(0,u),

where *u* is the input signal of the ReLU.

Following the convolutional and pooling layers, there are one or more fully connected layers which provide a convenient way of learning the combinations of the high-level features extracted by the previous layers and flatten the feature maps into a vector to facilitate the classification. The last layer in the CNN architecture is the output layer, which usually uses a Softmax function to give a probability distribution over the possible classification labels. The mathematical expression of the Softmax function is:

(3) $$P(y=j|z)=\;\frac{e^{z^{T}w_{j}}}{\sum {}_{q=1}^{Q}e^{z^{T}w_{q}}},$$

where **z** is the input vector of the output layer, which is usually the vectorized feature maps, *y* is the predicted label, *Q* is the number of candidate classes, and **w** consists the weighting parameters that can be obtained from model training.

### 2.2. Integration of PCA and SVM

In this section, for comparison with CNN, the commonly used PCA method [24] is integrated with the popular supervised learning technique, SVM [25], to achieve image-based flooding detection in packed columns. Here, the purpose of using PCA was to extract important features, reduce the dimensionality of the image data, and therefore reduce the computational burden of the following SVM-classifier training step. For simplicity, the method is denoted as PCA-SVM.

Suppose that the size of each image captured by the CCD camera is *I* × *J* and the number of total images used in model training is *K*. Accordingly, the image data can be stored in a three-dimensional matrix **X** with the size of *K* × *I* × *J*. Before conducting PCA, **X** is unfolded to a two-dimensional matrix **X** by merging the dimensions of the image size and keeping the dimension of the image number. Therefore, the size of **X** is *K* × *IJ*.

Then, the first principal component (PC) can be extracted by solving the following optimization problem:

$$\underset{p}{\max}{\left\|Xp\right\|}_{2}$$

(4) $$\mathrm{subject}\;\mathrm{to}\;{\left\|p\right\|}_{2}\leq 1,$$

where **p** is the loading vector with the dimensions *IJ* × 1, and ${\left\|\cdot \right\|}_{2}$ denotes the *L*_2_ norm. The first PC vector **t** can then be calculated as a linear combination of the columns of **X**, i.e., **t** = **Xp**, which explains as much variance in the data as possible. Further PC vectors, which are orthogonal to each other, are then obtained iteratively in a similar way by replacing **X** in Equation (4) by a deflated matrix [27,32].

As an alternative to solving the above optimization problem, the loadings and the PCs can also be obtained by conducting singular value decomposition (SVD) [25]. Usually, a small number of PCs are enough to extract a large portion of the variation information contained in the data, resulting in dimensionality reduction and feature extraction.

These PC vectors constitute a score matrix **T**, where each column of this matrix is a PC vector. Then, the score matrix **T** is used as the input of the SVM-classifier. For a test sample after PCA-based preprocessing denoted as *T_t_*, the prediction using the SVM-classifier *f*(*T_t_*) can be described as:

(5) $$f(T_{t})=\sum {}_{i=1}^{N_{s}}\alpha_{i}y_{i}k(T_{i},T_{t})+b,$$

where *T_i_*, *i* = 1, …, *N_s_* are the corresponding rows of the score matrix **T**; *N_s_* is the number of support vectors; $\alpha_{i}$ are the weights; $k(T_{i},T_{t})$ is the kernel function and the common Gaussian kernel form is utilized here, i.e., $k(T_{i},T_{t})=e^{-\left\|T_{i}-T_{t}\right\|/2\sigma^{2}}$ (σ>0 is the kernel width); *y_i_* is the label of the images; and *b* is the bias. The SVM-classifier has been adopted as a useful tool in pattern recognition and machine learning areas. Detailed algorithmic implements can be referred to [28].

### 2.3. Hyperparameter Selection

In this work, some hyperparameters were selected for model building, including the size and number *k* of the convolutional kernels in the CNN method, the embedding dimension of PCA in the PCA-SVM method, and the kernel width σ of the SVM classifier. Usually, the side length of a convolution kernel should be set to an odd number greater than one. In this work, the size of the convolution kernel was set to 5 × 5 according to the size of the input images and the suggestions in some related literature [33]. For the selection of *k*, there is no uniform formula. One common guideline is to ensure a high classification accuracy and a low computational load. The embedding dimension of PCA, i.e., the principal components retained in the model, can be set according to the explained variance by the model. In this work, the PCA model explained over 95% of the total variation information contained in the original image data. The kernel width σ of the SVM classifier was set according to 10-fold cross-validation [25].

## 3. Experimental System

**Figure 2:**
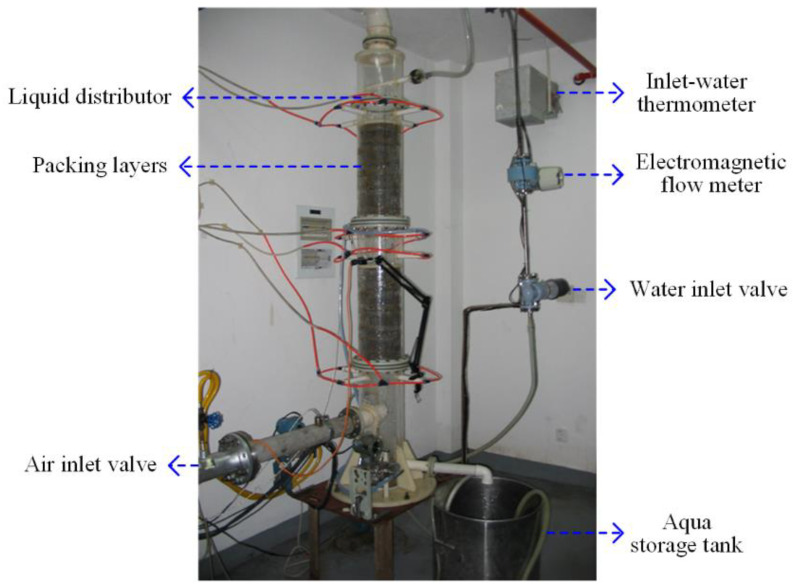
Packed column used in experiments.

**Figure 3:**
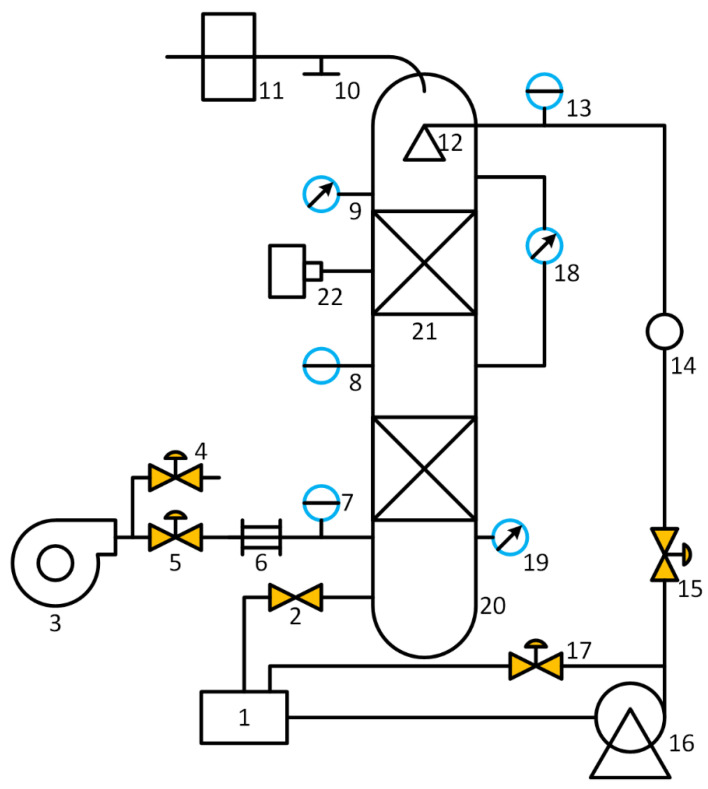
Schematic of experimental system (1. water tank, 2. drain valve, 3. centrifugal fan, 4. air bypass valve, 5. air inlet valve, 6. air flow meter, 7. inlet-air thermometer, 8. thermometer inside column, 9. pressure sensor in upper packing layer, 10. overflow outlet, 11. air vent, 12. liquid distributor, 13. inlet-water thermometer, 14. water flow meter, 15. water inlet valve, 16. water pump, 17. water bypass valve, 18. differential pressure sensor, 19. pressure sensor in lower packing layer, 20. packed column, 21. packing layers, and 22. digital camera).

In this section, the experimental system is introduced, which mainly consists of a packed column, a recycled air/water supply system, a process variable measurement system that is not used in this study, a digital camera, and a computer for data storage and processing. As shown in Figure 2, the cylinder of the packed column is made of transparent acrylic, facilitating the observation of the operation status inside the column [5]. The size parameters of the column are listed in Table 1. The structured packing is CY1700, a type of metal mesh corrugated packing material, whose geometer is described in Table 2. In the experiments, the ranges of air and water flowrates are 0–399 m^3^/h and 0–0.9 m^3^/h, respectively. A digital camera with charge couple device (CCD) sensors was chosen as the image-capturing device, which monitors the operation status in the upper packing layer. In the applications where the column is not transparent, the camera should be placed near the observation window on the wall of the column. The schematic of the experimental system is shown in Figure 3. A personal computer was used to store and process image data. The frequency for data collection was one frame per second.

The Matlab software was used to collect the 24-bit RGB color images whose resolution is 640 × 480 pixels. The main procedure of the experiments includes the following steps.

Step 1: Manipulate the water inlet valve to achieve a relatively large spray density. Adjust the air flowrate to a high value and let the packed column operate under a pre-flooding condition. Maintain the operation condition for more than 30 min to ensure that the packing material is sufficiently wet;

Step 2: Adjust the water inlet valve until a user-specified spray density is achieved. After that, increase the air flowrate slowly until the flooding phenomenon occurs. In the experiments, flooding is judged to occur by an experienced process engineer. Images reflecting the operation status are recorded periodically during the experiments. This step is repeated under various operating conditions to capture the images in both normal operation and flooding.

**Figure 4:**
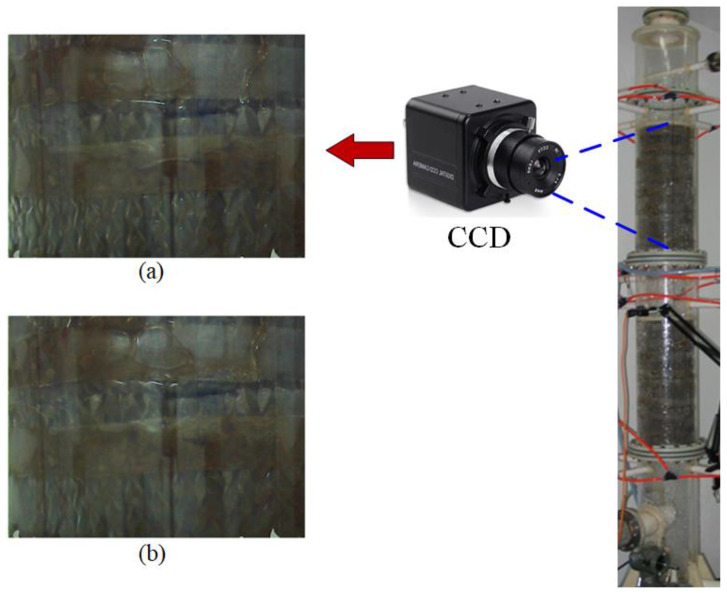
Image captured by the CCD camera from the observation window of the packing layer during (**a**) normal operation and (**b**) flooding.

Figure 4 shows some typical images recorded during the experiments. It is not easy to differentiate between these two situations by human eyes. Therefore, machine learning techniques should be implemented for applying computer vision in flooding detection.

## 4. Application Results

**Figure 5:**
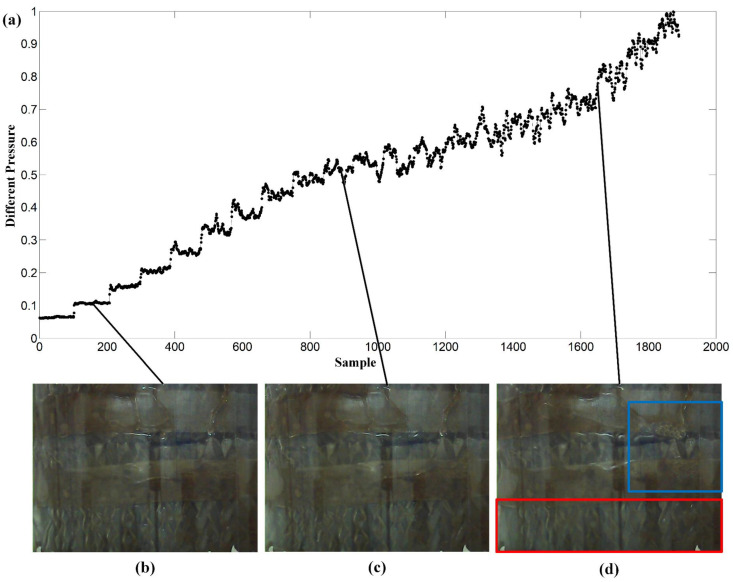
(**a**) The normalized trajectory of the pressure drop inside the packed column; (**b**) an operating condition that both air and water flowrates were low; (**c**) an operating condition when the pressure drop was increased and had a larger variation; and (**d**) a flooding phenomenon.

To ensure the effectiveness of the trained model in real-world applications, this study has conducted a design of experiments to stimulate the process dynamic characteristics. A broad range of data was collected during the operation of the packed tower, both in the present and future, with one image sampled per second, resulting in a total of 1890 images. This data set consisted of 1260 normal images and 630 flooding images, which included both non-flooded and heavily flooded states to provide a diverse training set and achieve high accuracy in prediction performance. In the experiments, the resolution of the original RGB images captured by the CCD camera is 480 × 640 pixels. To reduce the computational burden in the following model training steps, each image was downsampled to 120 × 160 pixels. Figure 5 shows the typical patterns in the images corresponding to different operating conditions, together with the normalized trajectory of the pressure drop inside the packed column. Figure 5b corresponds to an operating condition that both air and water flowrates were low. No flooding phenomenon is observed in this figure. In addition, the corresponding time series of pressure drop shown in Figure 5a is stationary. Both the mean value and variance are small. The image in Figure 5c was taken at another operating condition when the pressure drop was increased and had a larger variation. The variable trajectory became nonstationary. However, no significant flooding phenomenon can be identified in Figure 5c. In Figure 5d, the blue color boundary represents the occurrence of bubbles, and the red color boundary indicates the presence of entrainment, which are both signs of flooding in the packed column. At that condition, the pressure drop was high and varied dramatically.

**Figure 6:**
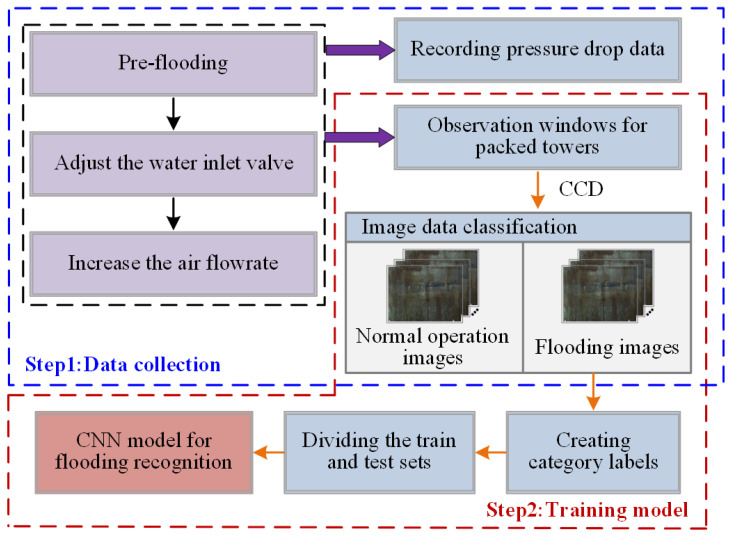
Flowchart of CNN-based machine vision method for detecting flooding.

To evaluate the proposed methods, the images were classified and labelled manually before the steps of model training. Based on the experience of process experts, all images collected during the experiments were divided into two classes corresponding to normal operation and flooding operation, respectively. Each image was labelled with a two-element row vector. For the images in Class I (i.e., the normal operation class), the label is [1 0]; while each image in Class II (i.e., the flooding operation class) has a label of [0 1]. Then, 300 of the total images (144 normal images and 156 flooding images, respectively), were randomly selected to make up the test set, while other images were used for model training. Figure 6 shows the flowchart of the CNN-based machine vision method for detecting flooding. This flowchart clearly outlines the steps involved in the implementation of our CNN-based machine vision approach for the non-destructive detection of flooding in packed columns.

The PCA-SVM model and the CNN model were trained based on the same training set. The data was standardized before model training. For the PCA-SVM method, the number of PCs was specified according to the explained variation. In detail, 200 PCs are selected, which explains more than 95% of the total variation contained in the image data. The parameters of the SVM-classifier are chosen using the common 10-fold cross-validation approach. The structure of the CNN is illustrated in Figure 1. As introduced, the input of the entire network is the three-channel RGB image whose height and width are 120 and 160, respectively. Therefore, it can be regarded as a volume with size of 120 × 160 × 3. The first convolutional layer (C1) has 10 kernels each of which has a size of 5 × 5 × 3. In other words, the size of the local receptive field is 5 × 5 and the information of all three channels is summarized. As a result, the size of the feature map generated by C1 is 116 × 156 × 10. Then, this feature map is inputted to the first pooling layer (S1). In this layer, the size of the filter is 4 × 4, while the stride is 4. Accordingly, the size of the output feature map is reduced to 29 × 39 × 10. The second convolutional layer (C2) includes 16 kernels with the size of 5 × 5 × 10 and generates a feature map with the size of 25 × 35 × 16. After another round of pooling (S2), the dimension of the feature map becomes 5 × 7 × 16. The filter used in S2 is of 5 × 5 and the stride is 5. The feature map outputted by S2 is then flattened and inputted to a fully connected layer (H) with 100 neurons. Finally, a 100 × 1 vector is inputted to the Softmax classifier to achieve the classification result. The weights used in different layers were obtained in model training by using the backpropagation algorithm [27]. The training parameters were set as follows: the batch size was set to 30, the learning rate was 1 × 10^−4^, and the Adam optimizer was chosen.

To further illustrate the advantages of the proposed method, the DBN method has been investigated for the non-destructive detection of flooding in packed towers. The DBN method is a representative deep learning approach that can extract nonlinear features from data using a general procedure [26]. This method has been successfully applied in industrial data analysis [34,35]. The DBN network structure consists of three layers of restricted Boltzmann machines and one layer of a backward propagation neural network. All methods were implemented with a computer configured running Windows 10 with an Intel i5-7300HQ, CPU at 2.5 GHz, and 16 GB RAM. The calculations were conducted with Python and MATLAB software. The hyperparameters settings for each method are described in Table 3.

**Figure 7:**
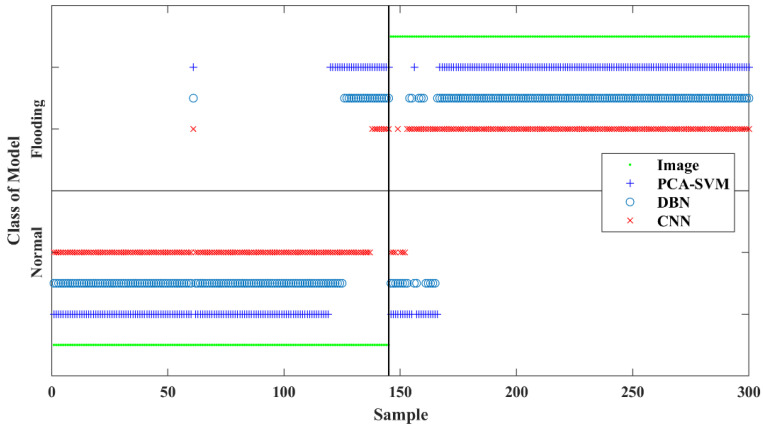
Classification results of the test images using CNN, DBN and PCA-SVM methods.

The main flooding recognition results of the test data are shown in Figure 7. The misclassifications mainly occurred to the image samples collected near the flooding point. The results demonstrated that the CNN method outperforms both the DBN method and the PCA-SVM method in terms. One main reason is that CNN is an end-to-end system for nonlinear pattern recognition. The inherent features in images can be extracted more efficiently. In comparison, PCA-SVM is a two-step indirect method. In addition, it should also be noted that the DBN method requires a longer training time on the same computing device as the other methods. From Figure 7, it is also observed that all models raise several false alarms just before the process reaching the flooding point. However, in the engineering viewpoint, this is not entirely a bad thing, because pre-alarms are desired for flooding prognosis. The operator can adjust the equipment operation parameters in time to ensure the safe operation of the packed tower. Additionally, because of the complex behaviour of fluid inside the column, the indications of flooding may appear at different locations in different images. In such cases, CNN outperforms PCA-SVM and DBN, due to its size and position invariance.

**Figure 8:**
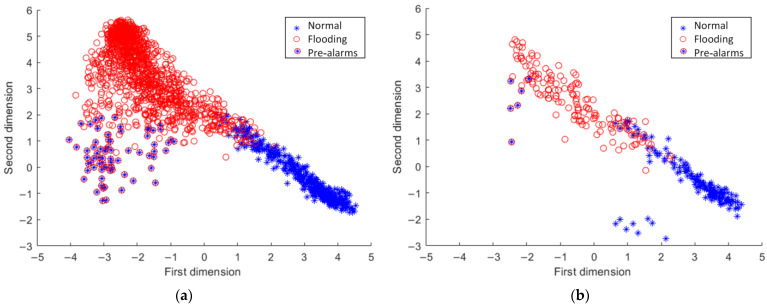
CNN hidden layer outputs of (**a**) training data and (**b**) test data.

To obtain a clear understanding of the reason of pre-alarms, the outputs of the CNN hidden layer are displayed in Figure 8, where each point corresponds to a sample, i.e., an image. Figure 8a represents the distribution of the training samples, while Figure 8b stands for the test samples. In these figures, it is clear that the images causing pre-alarms have different characteristics from those collected during both normal and flooding operations. Such results are understandable, because these images correspond to a transition period. Specifically, the images causing pre-alarms in the test set were much closer to the flooding cluster than the cluster of normal operation, indicating that the CNN model extract features related to flooding from these images. This is the reason why the CNN model identified them as flooding images, although the process engineers labeled them as normal operations.

To evaluate the classification performance quantitatively, four indicators, i.e., true positive (TP), true negative (TN), false positive (FP), and false negative (FN), are used. As listed in Table 4, TP and TN indicate that the classification is correct, while FP and FN mean the classification is incorrect. Table 5 lists the detailed classification results of different methods. In the test, CNN had more correct classifications (TP and TN) and fewer misclassifications (FP and FN). The classification accuracy of the CNN model was 95.33%, the PCA-SVM model was 84.67% and the DBN was 88.33%. This means that the CNN is superior to PCA-SVM and DBN in flooding recognition. Herein, to measure the likelihood of false and missed detection by the proposed model, in addition to the accuracy metric, the F1-score was introduced to assess the recall of the model for detecting flooding. The higher the F1 score, the better the classification performance of the model. The calculation results showed that the F1-score of CNN was 95.10%, which was much larger than the F1-score (83.69%) of the comparison method PCA-SVM and the F1-score (87.63%) of the DBN. Table 5 also lists the computational time of each method. It is worth mentioning that the test times in the table are for all test images. The comparison revealed that the computational time of CNN was between those of DBN and PCA-SVM methods. To conclude, taking into account the timely and accuracy of detecting flooding, the CNN method wins among the three.

Table 6 qualitatively concludes the advantages and disadvantages of the three models for the detection of flooding in packed columns. Taken together, it is concluded that CNN is applicable and advantageous in the task of detecting flooding. In the future, it will be important to make the model more robust to handle the presence of noise in images captured in high-noise industrial environments. To address this, data enhancement [36] will be an area of exploration. Data enhancement methods can reduce image noise and increase the number of small sample data, providing high-quality and diverse data for the model.

## 5. Conclusions

Real-time detection of flooding in packed columns is of the utmost importance for ensuring stable operation. With advancements in machine vision technology, automated visual inspection methods for non-destructive flooding detection are becoming increasingly viable. In this study, a deep learning machine vision approach based on CNN was proposed for non-destructive flooding detection. The performance of the CNN approach was compared with two representative methods, PCA-SVM and DBN. The experimental results, conducted on a real packed column, showed that the CNN method outperformed the other two methods. Compared to the PCA-SVM method, the CNN method improved accuracy by 12.59% and F1-score by 11.98%. The results also indicated that the CNN approach had fewer false alarms and missed alarms compared to the DBN method. For practical industrial applications, the packed column should be equipped with a viewing window and a CCD camera for real-time image capture. However, one limitation of the CNN approach is the high number of hyperparameters that need to be optimized based on the specific requirements. Future research may explore the use of metaheuristic algorithms or model augmentation strategies to address this challenge.

## Figures and Tables

**Table 1:** Size parameters of packed column.

Components of Packed Column	Size (m)
Cylinder diameter	0.22
Thickness of upper packing layer	0.46
Thickness of lower packing layer	0.46
Diameter of air inlet	0.09
Diameter of water inlet	0.02
Diameter of air outlet	0.11
Diameter of water outlet	0.05
Column height	2.20

**Table 2:** Geometry of CY1700.

Type	Material	Specific Surface Area (m^2^/m^3^)	Corrugation Angle (°)	Wave-Length (mm)	Unit Height (mm)	Porosity (%)	Range of Loading Rate (m^3^/(m^2^·h))
CY1700	Stainless steel	1700	45	3.2	100	85	7~24

**Table 3:** Hyperparameters for different models.

**PCA-SVM**	Penalty factor *C*	1		
	Kernel function	Radial basis function (RBF)		
	Gamma	1/2		
	Other parameters	Default		
**DBN**	Number of three hidden layer neurons.	[200 100 100]		
	Momentum	0.5		
	Max epoch	225		
	Batch size	1000		
	Penalty	2 × 10^3^		
	Learning rate	0.02		
	Activation function	Softmax		
**CNN**	Input images	120 × 160 × 3		
	Layer name	Type	Filter size, stride	Output size
	C1	Convolutional layer	5 × 5, 1	116 × 156 × 10
	S1	Pooling layer	4 × 4, 4	29 × 39 × 10
	C2	Convolutional layer	5 × 5, 1	25 × 35 × 16
	S2	Pooling layer	5 × 5, 5	5 × 7 × 16
	H1	Fully connected layer		100 × 1
	H2	Fully connected layer		2 × 1

**Table 4:** Possible states of prediction results.

		Actual Class	
		Normal (Positive)	Flooding (Negative)
Predicted class	Normal (True)	TP	FN
	Flooding (False)	FP	TN

**Table 5:** Quantitative performance comparison of different models.

	PCA-SVM	DBN	CNN
TP	118	124	136
TN	136	141	150
FP	20	20	6
FN	26	15	8
$\mathrm{Accuracy}(\%)=\frac{\mathrm{TP}+\mathrm{TN}}{\mathrm{TP}+\mathrm{FP}+\mathrm{TN}+\mathrm{FN}}$	84.67	88.33	95.33
$F1\text{-}\mathrm{score}(\%)=\frac{2\mathrm{TP}}{2\mathrm{TP}+\mathrm{FN}+\mathrm{FP}}$	83.69	87.63	95.10
Running time(s)	17.249 s (training) + 0.001 s (test)	366.290 s (training) + 0.040 s (test)	42.415 s (training) + 5.795 s (test)

**Table 6:** Qualitative comparison of the advantages and disadvantages of different methods.

	Advantages	Disadvantages
PCA-SVM	1. Improving generalization performance 2. Avoiding structural selection and local minima problems in neural networks	1. Sensitive to missing samples 2. Cumbersome to adjust parameters
DBN	1. Capable of reflecting the degree of similarity between similar data 2. No need to rely on empirical extraction of data features	1. Long training time 2. Easy to cause local optimal solutions
CNN	1. High parallel processing capability 2. High nonlinear feature extraction ability 3. Noise-insensitive and highly robust	1. Timely model updates for application 2. Requires a large number of parameters

## Data Availability

The data presented in this study are available on request from the corresponding author.
